# Inactivation of *Pichia rhodanensis* in relation to membrane and intracellular compounds due to microchip pulsed electric field (MPEF) treatment

**DOI:** 10.1371/journal.pone.0198467

**Published:** 2018-06-25

**Authors:** Ning Zhu, Ning Yu, Yue Zhu, Yulong Wei, Haiping Zhang, Ai-dong Sun

**Affiliations:** Department of Food Science and Engineering, College of Biological Sciences and Technology, Beijing Forestry University, Beijing, China; University of Campinas, BRAZIL

## Abstract

The effect of microchip pulsed electric field (MPEF) treatment on lethal and sublethal injury of *Pichia rhodanensis (P*. *rhodanensis)* were employed under 100–500 V for 20–100 pulses and the underlying mechanism of MPEF treatment was investigated as well. A 6.48 log_10_ reduction of *P*. *rhodanensis* was achieved at 500V for 80 pulse. The fluorescent staining with Propidium Iodide (PI) verified that the rate of sublethal injury cells maximum up to 27.2% under 200 V. MPEF can cause the damage of cell morphology and ultrastructure, meanwhile causing a decrease in cellular enzymes, antioxidant enzyme activity and cell membrane fluidity. The leakage of intracellular compounds (protein, nucleic acid, K^+^, Mg^2+^) and Ca^2+^-ATPase gradually increased as the growth of voltage, especially the proportion of protein in the supernatants increased from 2.0% to 26.4%. Flow cytometry analysis showed that MPEF has significant effect on membrane potential, but no obvious influence on non-specific esterase. MPEF can cause the changing of the secondary structure of protein, at the same time, double helix structure of DNA became loose and unwinding. These results provide a theoretical guidance for the widespread using of MPEF technology in the application of a non-thermal processing technique for food.

## Introduction

Pulsed electric field (PEF) technology is one of the most popular non-thermal food sterilization technology in the world [1]. The results of some studies show that PEF can effectively inactivate microorganisms at mild temperature [2–4]. However, the strong electric field is generated by a relatively high voltage [5, 6]. This process leads to high costs and difficult to manipulate. Therefore, effective sterilization at low voltage while avoiding shortcomings of traditional processing chamber has become a popular research topic in PEF.

With the development of microfabrication, wherein the space between two electrodes is short, low voltage can produce high electric field strength. To date, several laboratories have developed microchips with germicidal function [7]. However, little is known about the effect of MPEF on inactivating microorganisms, let alone mechanism of microbial inactivation. There are some hypotheses about the mechanism of microbial inactivation under PEF, in which two models of electrical breakdown [8] and electroporation [9] are generally accepted, the cell membrane damage and intracellular compounds leakage induced by PEF are related to microbial inactivation [10, 11]. Studies illustrated that pores caused by PEF in membrane could be reversible or irreversible [12]. Reversible pores result in sublethal injuries, while irreversible pores lead to the cell death [13]. Previous studies of sublethal injuries and cell structure damage are mainly through selective media [14], scanning electron microscopy (SEM) and transmission electron microscopy (TEM) [15]. Flow cytometry (FCM) [16] in combination with fluorescent techniques offers a powerful tool for real-time data acquisition and quantitative analysis of analyzing a cell populations at the single-cell level, which could observe changes in specific cellular components, such as the membrane, nucleic acid, non-specific esterase and membrane potential [17–19]. Poor cell membrane fluidity and increased leakage of intracellular compounds with increasing PEF treatment were also illustrated [11, 20]. In addition, superoxide dismutase (SOD), catalase (CAT) and glutathione peroxidase (GSH-Px) are basic antioxidant enzymes, having an irreplaceable regulatory effect on the life activities of microorganisms [21]. K ^+^ and Mg^2 +^ have important significance to maintain the normal osmotic pressure of cells [22]. However, detailed aspects about the influence of PEF to these changes are still far from clear. At present, the knowledge on MPEF inactivation effect of microorganisms and its mechanism are limited. Although it belongs to electric field processing, as same as PEF, whether the micro-treatment chamber will have different effects on microorganisms need to be studied.

*Pichia rhodanensis (P*. *rhodanensis)* is a common microorganism that causes fruit juice spoilage. In this work, *P*. *rhodanensis* was selected as a model to assess MPEF induced lethal and sublethal cellular damage at different voltage by selective media and Propidium Iodide (PI) staining techniques. In addition, the underlying mechanism of MPEF treatment to *P*. *rhodanensis* inactivation was explored, mainly focusing on the leakage of intracellular compounds and changes of morphology, membrane fluidity, cellular enzymes, proteins, nucleic acids and membrane potential induced by MPEF. The objective of this study is to obtain more information on the microbial damage caused by MPEF. Moreover, the information would be useful in defining adequate MPEF treatments to assure food stability and safety.

## Materials and methods

### Preparation of cell suspension samples

*P*. *rhodanensis* (China General Microbiological Culture Collection Center, CGMCC, 2.2376) was maintained on slants of Yeast Extract Peptone Dextrose (YPD) agar medium (Aobo Star Biotechnology Co., Ltd., Beijing, China), one single colony was inoculated from the YPD agar medium into a cone bottle with 50 mL of sterile YPD broth medium, and then incubated at 32 °C in a shaker (150 rpm) for 12 h. Cells were centrifuged (3H16RI Refrigerated Centrifuge, herexi, China, 7000 rpm, 4°C) for 5 min, and then re-suspended in sterile phosphate buffer (PBS, 10 mM, pH 7.0). Finally, 50 mL of the cell suspension with a concentration of 106–10^7^ CFU/mL was treated by MPEF.

#### MPEF treatment system

**Fig 1:**
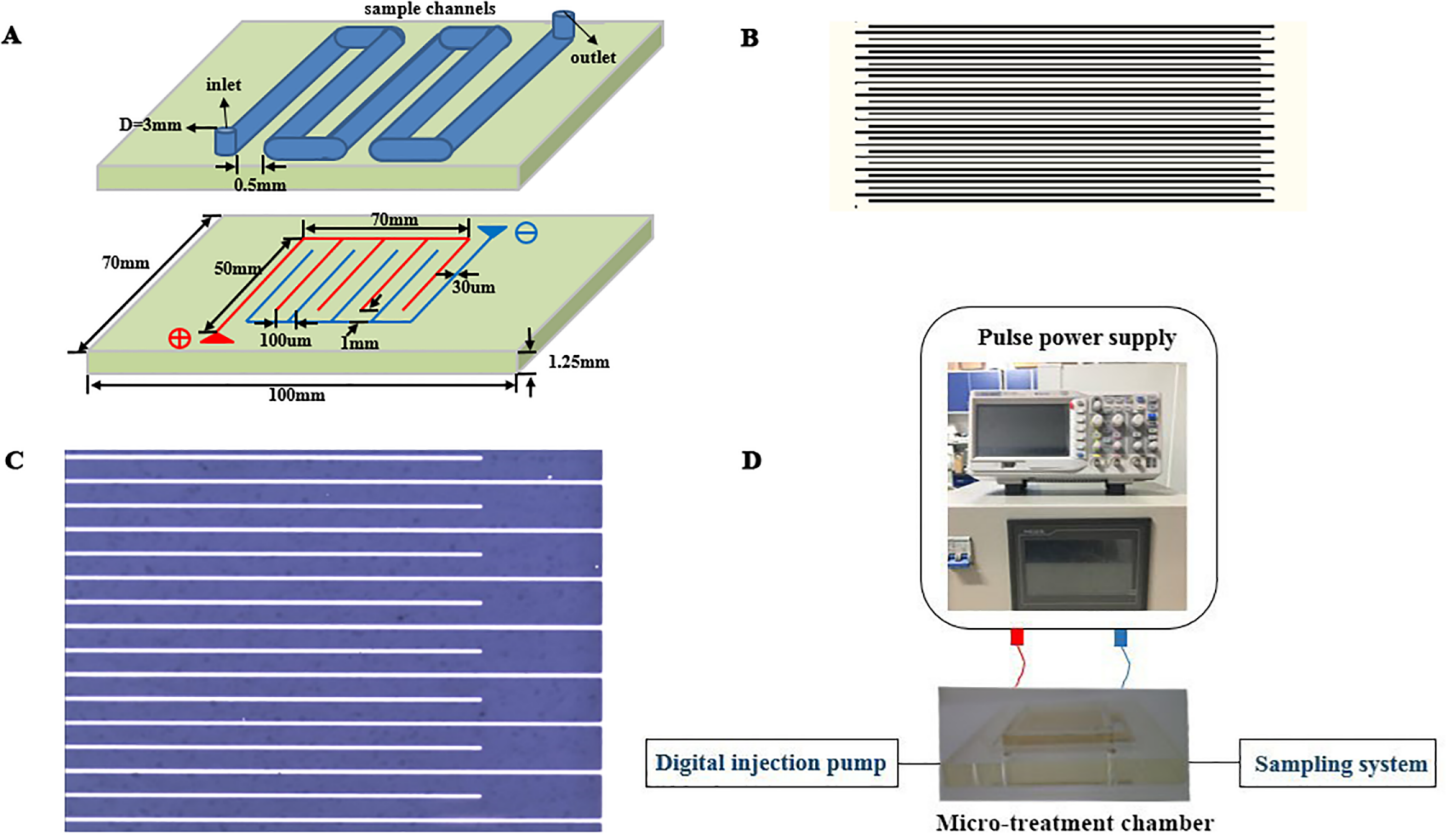
Schematic of the experimental setup. (A) Schematic of the microchip with the detailed topology parameters. The multi-electrode array (red and blue lines) was etched on glass basement membrane, and sample channels were etched on the PDMS and set on the top of electrode. (B) Partial enlargement of microchip by CAD. (C) Partial enlargement of microchip by microscope. (D) Schematic of the experimental setup, consisting of Pulse power supply, Oscilloscope, Micro-treatment chamber, Digital injection pump and Sampling system.

In this section, a laboratory-scale, continuous MPEF treatment system consisting of customized pulse power equipment (Suo Yi Electronic Technology Co., Ltd., Shanghai, China) with square wave (frequency: 120Hz, pulse width: 200μS, pulse front edge ≤ 150nS) and self-designed microchip [23] are proposed as shown in Fig 1A with the corresponding partial enlargement shown in CAD (Fig 1B) and microscope (Fig 1C). Microelectrode comprises two-layer structure, including insulating glass basement membrane and multi-electrode array (gold film is plated on the copper layer) which is composed of positive (red line) and the negative (blue line) arrays. The channels were placed above the electrode array as shown in Fig 1A, the electrode spacing of the microchip was 100 μm, and the sample channels (3 mm) was etched on the PDMS and set on the top of electrode. The schematic of the experimental setup is shown in Fig 1D. It contains of pulse power supply system, oscilloscope, micro-treatment chamber, digital injection pump and sampling system. Pulse power supply system is connected to micro-treatment chamber by positive and negative wires to provide pulse voltage, digital injection pump and sampling system are linked to the two opposite sides of micro-treatment chamber to control flow velocity of juices. The pulse width was set at 0.20 ms, interval time was 8 ms. Before and after each treatment, the MPEF system was cleaned and disinfected with 75% (v/v) ethanol: water solution, and then rinsed with sterile distilled water.

#### Detection of inactivation and sublethal injury by plate count

The non-selective medium is YPD agar medium, the medium supplemented with NaCl is used as selective medium, and the corresponding concentrations is 4.5% (w/v) NaCl: YPD agar medium. The influence of MPEF on inactivating *P*. *rhodanensis* was studied by comparing the logarithmic decrease at different voltages (100—500V) and pulse numbers (20–100). The logarithm value (lgS) [24] was calculated as follows:

$$lg\;S=lg\left(N_{0}/N_{1}\right),$$

where *N*_0_ is the number of microorganisms surviving on the non-selective or selective medium before treatment (CFU/mL), and *N*_*1*_ is the number of microorganisms surviving on the homologous medium after treatment (CFU/mL).

#### Fluorescent staining and flow cytometry (FCM) analysis

PI (Sigma) is a membrane impermeable fluorescent dye, only when the cell membrane is subjected to a certain degree of damage can PI enter into the defective cells and bind to nucleic acid [25]. Cells were incubated with 50 μL PI (0.1 mg/mL sterile water) for 10 min at 4°C before and after MPEF treatments.

Carboxyfluorescein diacetate (CFDA, Sigma) can penetrate cell membrane to detect the changes of intracellular non-specific esterase activity [26]. Untreated and MPEF-treated cells were incubated with 50 mM CFDA at 37°C for 10 min.

Acridine orange (AO, Sigma) can enter into intact membranes, it produces green fluorescence when bound to double-stranded DNA and red fluorescence with single-stranded nucleic acid [27]. Untreated and MPEF-treated cells were incubated with 100 μL AO (0.1 mg/mL sterile water) at 37°C for 15 min. RNase-treated group were incubated with 10^3^ units/mL RNase [28] at 37°C for 30 min before AO staining.

Rhodamine 123 (RH123, Sigma) is a membrane permeable fluorescent dye, yellow-green fluorescence of cells will decline with the decreasing of mitochondrial membrane potential [29]. Untreated and MPEF-treated cells were incubated with 100 μL RH123 (0.1 mg/mL sterile water) at 30°C for 10 min to indicate the changes of transmembrane potential.

The above-stained cells were washed twice with PBS to remove excessive dye, and then filtrated with a 300 copper mesh screen, followed by BD LSRFortessa and BD FACSCalibur flow cytometer (Becton, Dickinson and Company, USA). About 10,000 cells were analyzed for each sample after excitation with a 488 nm argon laser, and delivered at the low flow rate of 400–600 cells per seconds.

#### Determination of cytomembrane fluidity

1,6-diphenyl-1,3,5-hexatriene (DPH, Sigma-Aldrich St. Louis, MO) was used as the fluorescent probe to monitor the changes in membrane fluidity [30] of *P*.*rhodanensis* after MPEF treatment. The thallus were resuspended in 4.0 mL of 2.0 μM DPH solution and incubated at 37 °C for 1 h, and then centrifuged (7000 rpm, 5min) and washed 3 times by sterile PBS buffer. The fluorescence anisotropy was determined by spectro-fluorometer (F-7000, Hitachi, Japan). Determinations were performed at excitation and emission wavelengths of 358 and 429 nm using 5.0 nm slit width. Fluorescence polarization and its anisotropy were calculated as follows,

$$P=\frac{I_{VV}-{GI}_{VH}}{I_{VV}+{GI}_{VH}}$$

$$\gamma =\frac{I_{VV}-{GI}_{VH}}{I_{VV}+{2GI}_{VH}}$$

$$\eta =\frac{2P}{0.46-P}$$

where P, γ, η and G represent fluorescence polarization, anisotropy, microviscosity and instrument grating factor, respectively. I_VV_ and I_VH_ denote fluorescence intensities of emission polarizer vertically and horizontally when the excitation polarizer is oriented vertically [31].

#### Electron microscopic analysis

S-3400N scanning electron microscope (Hitachi, Japan) and H-7650B transmission electron microscope (Hitachi, Japan) were used to observe the surface and ultrastructure changes of cells after the MPEF treatments for 0 V, 200 V and 400 V. The specific sample preparation method was referred to that of Machado [32] and Moody [33].

#### Assay of cellular enzymes activities

Ca^2+^-ATPase assay kit (Genmed Scientifics Inc., USA) were used to measure Ca^2+^-ATPase activities according to the manufacturer’s protocol. The intracellular enzymes activities with and without MPEF treatment were determined by an APIZYM kit (BioMérieux Co, France) monitoring 19 enzymatic activities from a complex system. Substrates were mixed with the cell suspension and incubated at 37 °C for 4 h, and colors were developed by adding reagent of ZYMA and AYMB [34]. The color changes were measured by a UV-mini-1240 UV spectrophotometer (Shimadzu, Japan), and enzyme activities were expressed as a percentage of color changes.

SOD, Catalase and GSH-Px Assay Kit (Sigma) were used to detect the changes of SOD, CAT and GSH-Px activities after MPEF treatment. All results were expressed as relative enzyme activity (R%),

$$R\%=\frac{A}{A_{0}}$$

where A represents the enzyme activity of MPEF-treated samples, A_0_ represents the enzyme activity of samples without MPEF treatment.

#### Protein and nucleic acid structure analysis

Total proteins of cells were extracted using the Yeast Protein Extraction Kit (BIO-RAD Co, USA). SDS-PAGE was performed using 4% stacking gel and 12% separating gel, and samples were mixed at a 1:2 ratio with the reductive sample buffer. 20 μL sample solution was added to the sample hole.

Circular dichroism spectra were collected in the far-UV range (197–260 nm) by J-720 CD spectropolarimeter (JASCO, Japan) with a quartz cuvette of 1 mm optical path length. The samples were scanned at the rate of 50 nm/min with 0.1 nm bandwidth in triplicate. The secondary structures of *P*.*rhodanensis* proteins with and without MPEF treatment were analyzed. The changes in nucleic acid structure were determined under the same conditions with near-UV CD spectra (250–320 nm) [35].

#### Measurement of MPEF on content leakage

*P*.*rhodanensis* cells suspensions with and without MPEF treatment were centrifuged at 7000 rpm for 5 min. The absorbance of the supernatant at 260 nm and 280 nm were determined by UV absorption method [36] to investigate the leakage of nucleic acids and proteins. The leakage of K^+^ and Mg^2+^ were determined by atomic absorption spectrometry [37]. BCA Protein Assay Kit (Tiangen Biotechnology Co., Ltd., China) was used to determine the concentration of protein.

#### Statistical analysis

All measurements were performed in triplicate. Data were compared by analysis of variance (ANOVA) using the Statistical Program for Social Sciences (SPSS) software (version 16.0) with a significance level of P ≤ 0.05. The statistical analyzes were implemented by Origin 9.0 software.

## Results and discussion

### Effect of MPEF on the lethal and sublethal injury of *P*. *rhodanensis*

**Fig 2:**
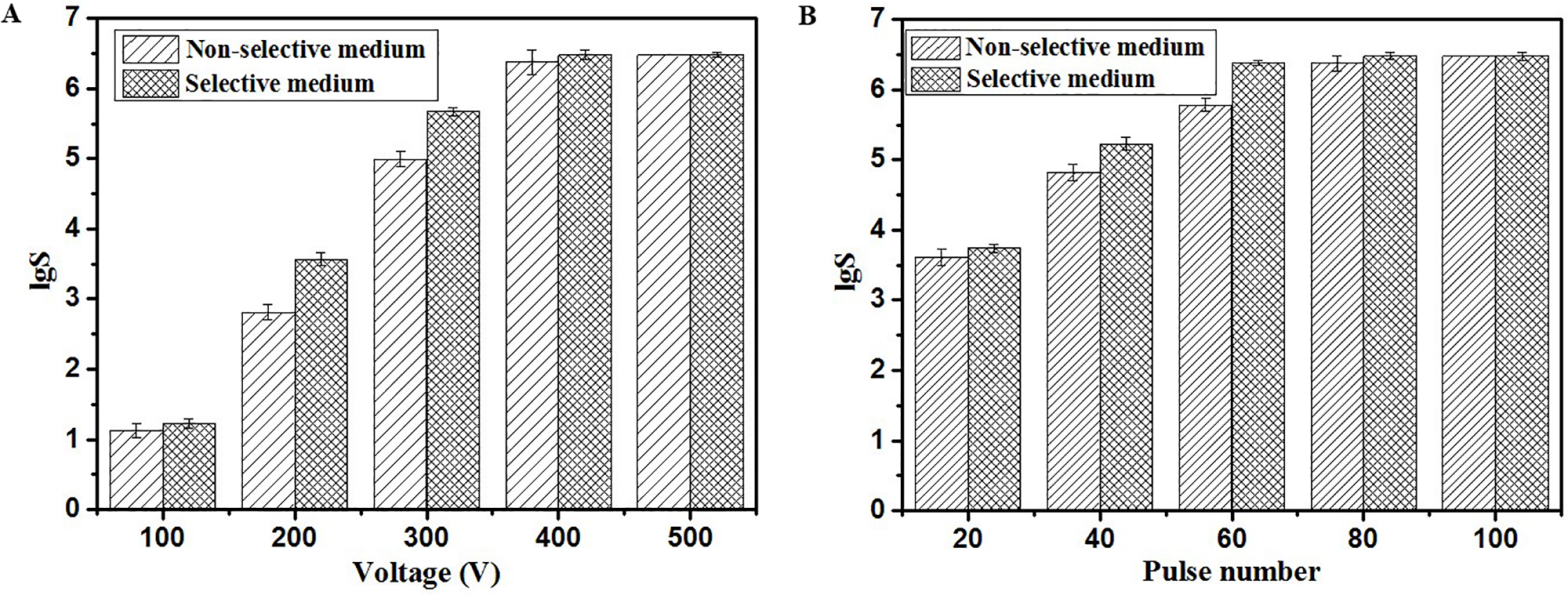
Logarithm decline of *P*. *rhodanensis* in the non-selective (Histogram with diagonals) and the selective medium (Histogram with cross diagonals) with respect to the voltage (A) and pulse number (B). 120Hz square wave, 0.20 ms pulse width.

The inactivation of *P*. *rhodanensis* after MPEF at different voltage and pulse number followed by plating onto selective medium (SM) and non-selective medium (NM) were first explored (Fig 2), both undamaged cells and sublethally damaged cells could grow at NM, while only the undamaged cells could survive at the SM [38]. As shown in Fig 2, a higher inactivation effect was achieved for *P*. *rhodanensis* under the same voltage and pulse number when cultivated in the SM. Especially a 3.56±0.09 log_10_ cycles reduction of *P*. *rhodanensis* cultivated in SM at 200V for 80 pulse number, higher than 2.81±0.11 log10 cycles reduction cultivated in NM. The quantity variance after MPEF treatment in different medium indicated the presence of sublethally injured cells. Wang et al. [39] reported a similar result that PEF can cause *Saccharomyces cerevisiae* intact, sublethally injured or dead. As shown in Fig 2A, in general, increasing the voltage from 100V to 500V had a significant effect (*P* < 0.05) in reducing microbial counts, 1.12±0.10 to 6.48±0.00 log_10_ cycles at NM and 1.23±0.07 to 6.48± 0.03 log_10_ cycles at SM. With the voltage extending from 100V to 200V, the sublethally injured cells increased rapidly, at this point, continuously increasing the voltage, these cells have more likelihood of being irreversible damage, therefore, the number of sublethally injured cells was declined. However, the decline in microbial counts and sublethally injured cells counts have no significant change when voltage was increased from 400 V to 500 V, representing that 400V can be sufficient for the inactivation of *P*. *rhodanensis* and this damage was irreversible. Therefore, 400V was maintained to carry out subsequent studies, and pulse number showed similar MPEF resistance. Compared with the voltage, it had a slight effect on inactivating *P*. *rhodanensis*, whether this effect is reversible or irreversible. As shown in Fig 2B, a high decline in microbial counts (6.37±0.11) and less sublethally injured cells counts were obtained when the cells were treated by MPEF with 80 pulses. Therefore, 80 pulses at 400V were adequate to achieve inactivation of *P*. *rhodanensis*. Similarly, PEF has significant impact on yeast, gram-negative, gram-positive cell in lethal and sublethal injury [40, 41].

**Fig 3:**
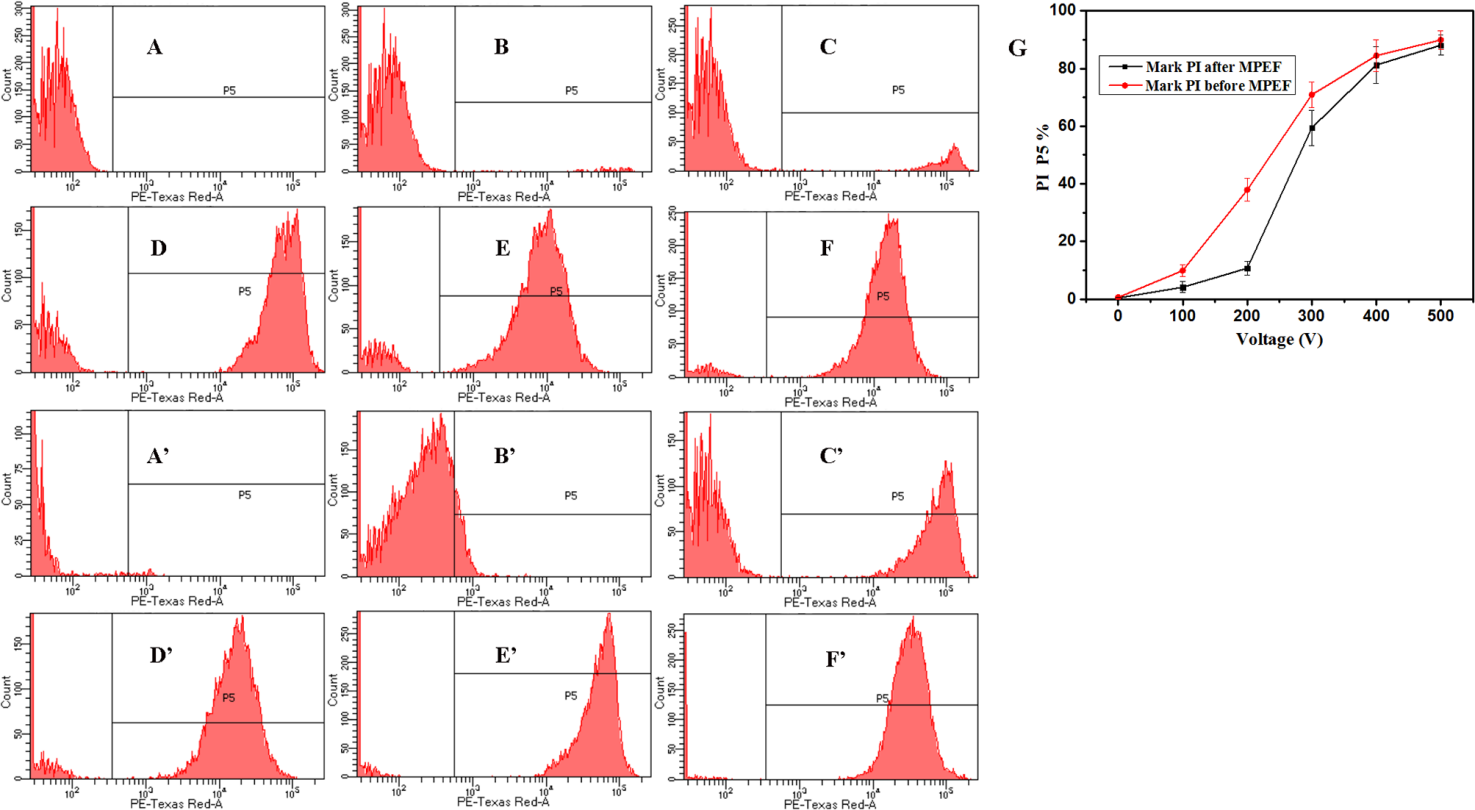
The PE-Texas Red-A (red fluorescence collected at 630 nm) channel fluorescence histograms of *P*. *rhodanensis* stained by PI.

In order to address sublethal injury/voltage relationship more precise, the fluorescent staining with PI was used. The cells marked by PI demonstrating the incomplete membrane [42]. Flow cytometry histograms of *P*. *rhodanensis* cells stained with PI before and after MPEF to evaluate the reversible and irreversible membrane damage (Fig 3). Cells in P5 region are marked by PI, others are autofluorescence. Fig 3 displays the percentage of PI-stained cells exposed to MPEF under the two labeling methods. Most of the untreated cells (A, A’) were PI negative, indicating the integrity of membrane. The PI fluorescent events increased with the arising of voltage from 100 V to 500 V (B-F, B’-F’), this means more and more damage occurred in cell membrane. P5% stands for the percentage of PI-stained cells in Fig 3G. There was a higher P5% when the dye was added before the MPEF treatment. In especial, after 200 V and 80 pulses, P5% was 37.9±4.0 when PI was added before MPEF treatment, however, it was only 10.7±2.3 when it was added after MPEF treatment. The difference of P5% under the same MPEF treatment conditions reveals the existence of sublethal injury cells [43]. Therefore, the rate of sublethal injury cells was maximum up to 27.2%, which is slightly higher than *Escherichia coli* (*E*.*coli*, approximately 20%) after PEF treatment [44]. The existence of sublethal injury cells could be explained by the lower voltage causes reversible electroporation in the cells, if the voltage is higher than the critical value, the electroporation is irreversible [45]. It is noticeable that this study obtained similar results of plate count (Fig 2).

The cells were exposed to MPEF treatments at 80 pulses for 0 (A, A’), 100 (B, B’), 200 (C, C’), 300 (D, D’), 400 (E, E’), 500 (F, F’) V. A-F represents the cells were marked PI after MPEF, A’-F’ represents the cells were marked PI before MPEF. (G) Percentage of *P*. *rhodanensis* cells in P5 after MPEF in PBS at 80 pulses for 0-500V, the red line represent PI is marked before MPEF and the black line represent PI is marked after MPEF.

### Effect of MPEF on the morphology and membrane fluidity of *P*. *rhodanensis*

**Fig 4:**
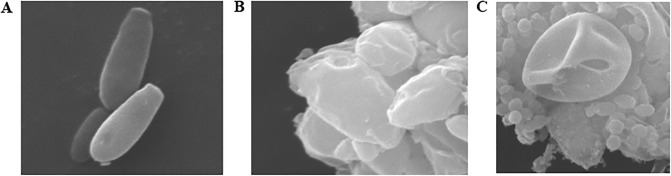
SEM images of the *P*. *rhodanensis* cells: (A) untreated cells; MPEF-treated cells: (B) 200V, (C) 400V.

Fig 4 showed the morphology of *P*. *rhodanensis* cells by SEM, and the healthy cells topology (Fig 4A) without any treatment was observed as a smooth and continuous cell surface. In addition, a spot of birth scars and bud scars were also observed. Compared with these cells, the MPEF-treated cells showed visible changes in their morphology only after 200V (Fig 4B). With the increasing in birth scars and bud scars, cells surface appeared wrinkles and become roughness. The above phenomenons were intensified when 400V was applied (Fig 4C), higher damage was observed in the cells treated with higher voltage. There were obvious holes on the surface and a lot of cytoplasmic leakage. Machado et al. [32] found out that the damage of cell membrane is a possible cause for the *E*.*coli* death. Our results indicated that the alteration may occurred in the cells surface when subjected to MPEF, it is possible to conclude that there is a connection between cell death and damages in the cell morphology. The structural difference of strains leads to different treatment conditions [34, 46] to achieve the same degree of damage.

**Fig 5:**
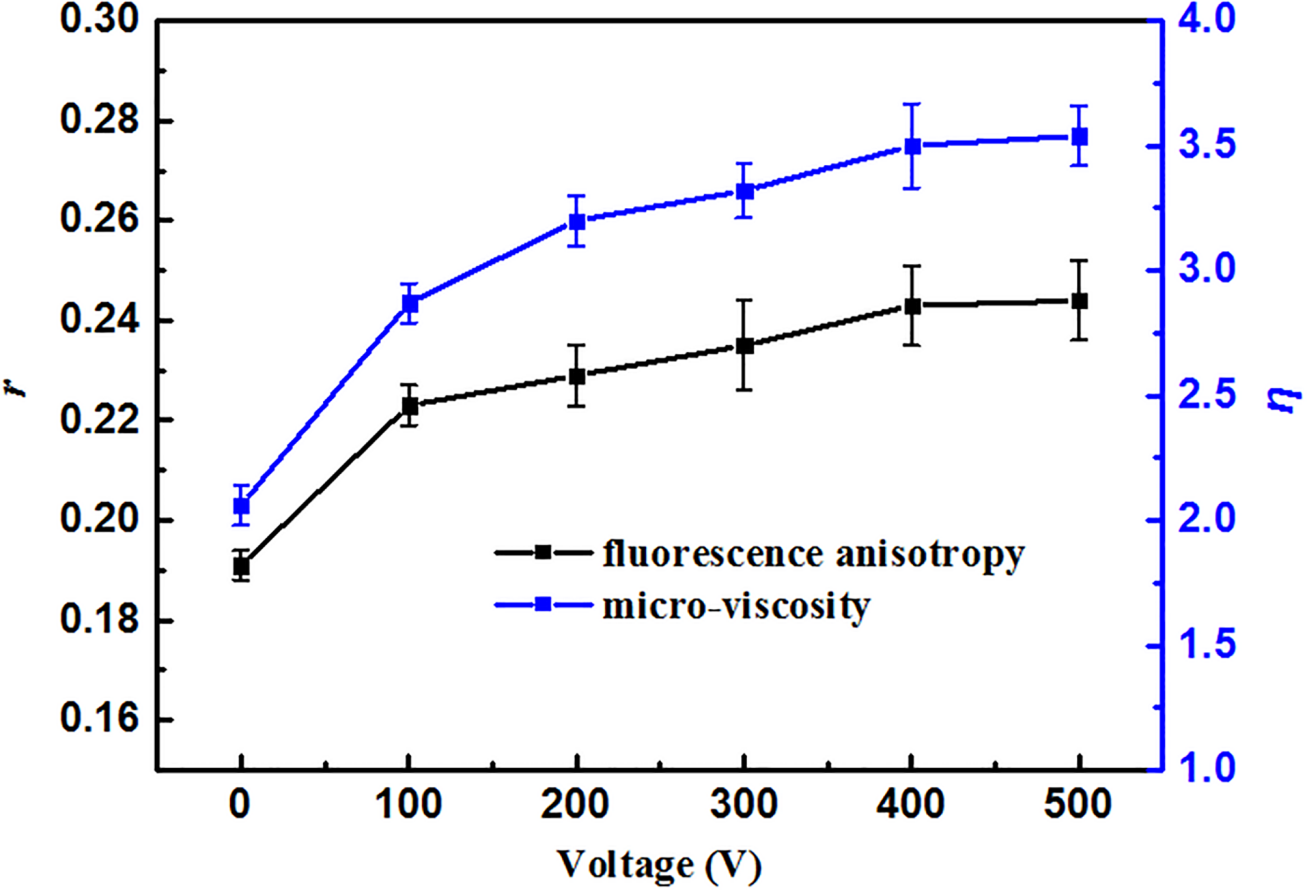
The change of fluorescence anisotropy (γ) and micro-viscosity (η) of *P*. *rhodanensis* cell membrane at different voltage.

Fig 5 showed the effect of voltage on *P*. *rhodanensis* membrane fluidity. The probe polarization ratio and cytoplasmic membrane fluidity are inversely correlated [47]. The values of fluorescence anisotropy (γ) dramatically increased (form 0.19±0.003 to 0.24±0.008) as the growth voltage from 0V to 400V, but the change was not significant in the course of voltage increased from 400V to 500V. Similar results were obtained from the change in micro-viscosity (η) values of *P*. *rhodanensis* cells compared to the untreated cells, approximately 1.75-fold increase in η value was obtained in cells treated at 400V. These results demonstrate a significantly decrease in membrane fluidity, which has a good correlation with cell death.

### Effect of MPEF on the content leakage of *P*. *rhodanensis*

**Fig 6:**
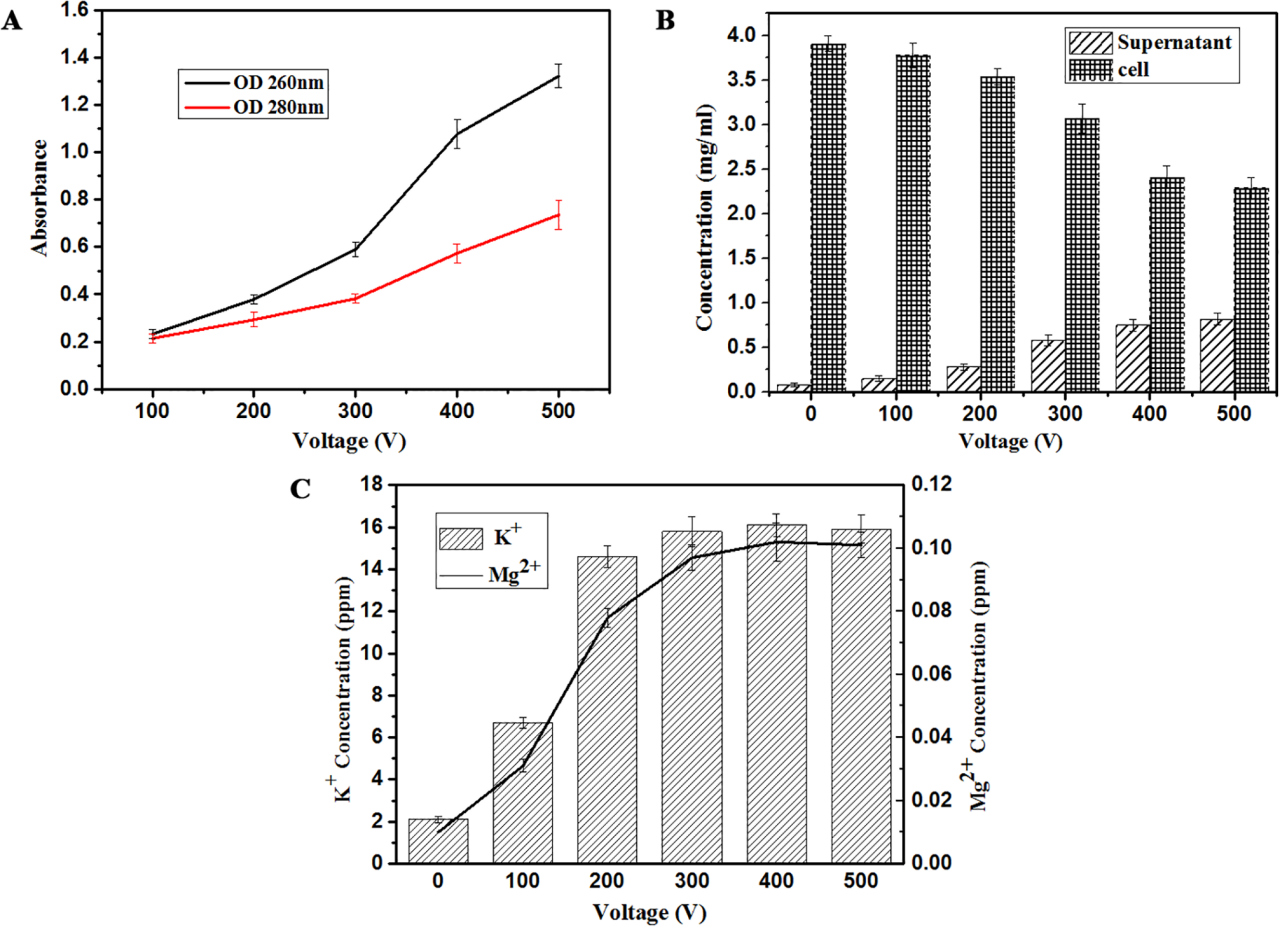
Leakage of intracellular substances from *P*. *rhodanensis* after MPEF treatment at different voltage. (A) Protein (OD 280nm) and nucleic acid (OD 260nm) leakages from *P*. *rhodanensis* after MPEF treatment at different voltage. (B) Protein concentration in the supernatant (Histogram with diagonals) and cell (Histogram with cross lines) from *P*. *rhodanensis* after MPEF treatment at different voltage. (C) K ^+^ (Histogram with diagonals) and Mg^2 +^ (Broken line) leakage from *P*. *rhodanensis* after MPEF at different voltage.

There were protein and nucleic acid leakage happened of *P*. *rhodanensis* after MPEF treatment (Fig 6A). The changed trends of these two substances were same, and massive leakages of them were obtained from 300V to 500V. These may be due to the increase of cell membrane permeability leaded by MPEF, which is related to cell death. PEF treatment also has a similar phenomenon [48]. For the sake of quantifing the effect of MPEF on protein leakage, a study was performed to determine the influence of voltage on protein concentration in the supernatant and cell (Fig 6B). The mass concentration of protein in the cells and supernatants without MPEF treatment were 3.91 mg/mL ± 0.09 and 0.08 mg/mL ± 0.02, respectively, 98.0% and 2.0% of the total. Results showed that the increase of voltage increased linearly the supernatant protein concentration, on the contrary, the protein concentration in the cells were gradually decreased. When the 500V was applied to them, the protein in cells and supernatants accounted for 73.6% and 26.4%. The changes are smaller than that of *S*. *cerevisiae* after PEF treatment [49]. In the present study as shown in Fig 6C, the ion (K ^+^, Mg^2+^) leakage was assessed after MPEF at different voltage. The K ^+^ leakage of the *P*. *rhodanensis* increased as a function of voltage. MPEF treatment from 100V to 200V resulted in the most rapid release of ions. Obviously, the increase of Mg^2+^ in medium showed the same trend as K ^+^ leaking. In contrast to K ^+^, the concentration of Mg^2+^ in medium was much less, although the levels of Mg^2+^ in medium also increased significantly in response to MPEF treatment. Their leaks mean that the cells are in an abnormal osmotic state, causing cell death.

### Effect of MPEF on the activity of cellular enzymes of *P*. *rhodanensis*

**Fig 7:**
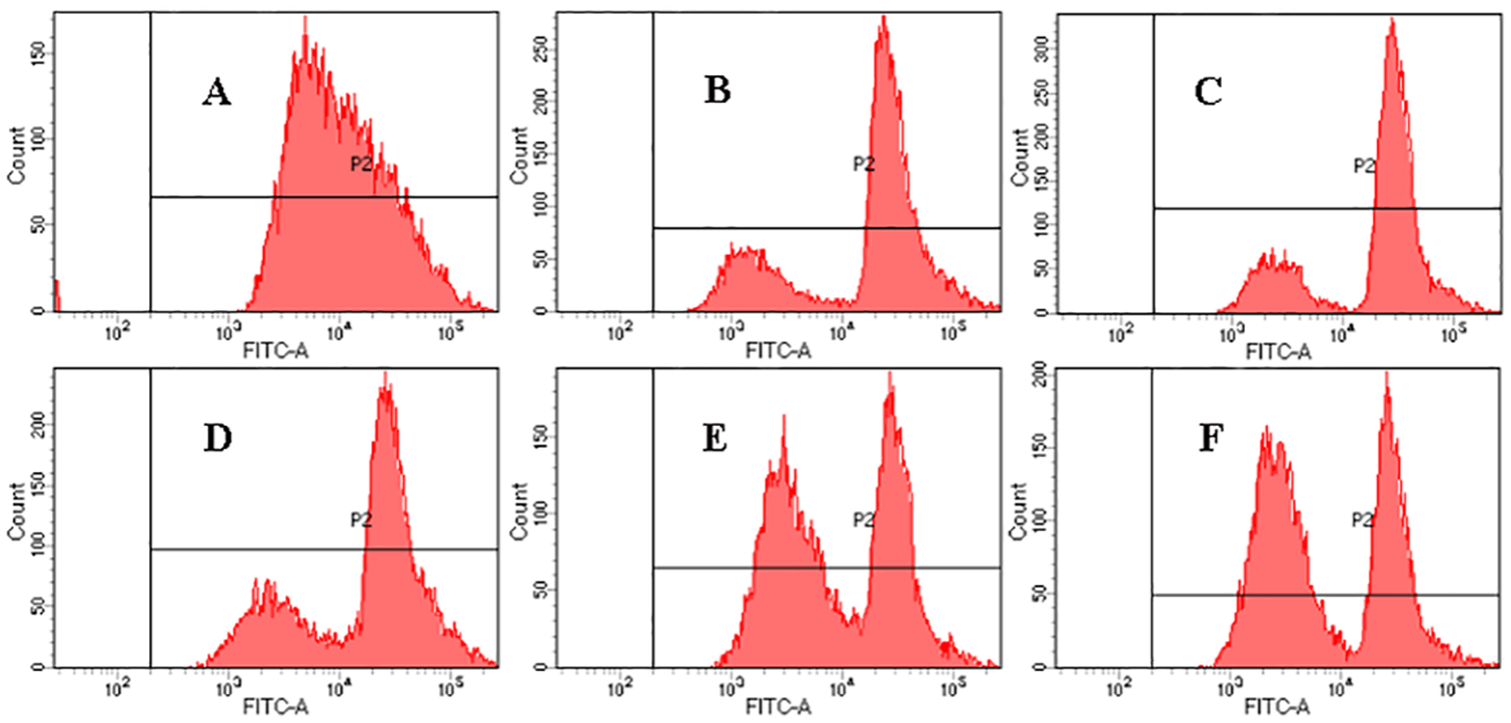
The FITC-A (green fluorescence collected at 525 nm) channel fluorescence histograms of *P*. *rhodanensis* stained by CFDA.

The CF-stained combined with FCM analysis were used to estimate the non-specific esterase activity [50], and the fluorescence histograms of *P*. *rhodanensis* stained by CFDA after MPEF treatment under 0-500V could be found in Fig 7. Cells in P2 region are marked by CFDA, others are autofluorescence. P2% and fluorescence intensity mean (M) value were shown in Table 1, the most of untreated *P*. *rhodanensis* cells were CF-stained (99.7%), whereas all the MPEF-treated cells were CF-stained (100%). The M value of MPEF-treated cells increased firstly (100V-300V) and then decreased (300V-500V), but still higher than that of untreated group, the increase of M value means higher non-specific esterase activity of *P*. *rhodanensis* [51]. In addition, MPEF-treated cells showed bimodal peaks, demonstrating the appearance of two size cells [52]. These results can be explained by that electric field accelerates the production of budding cells, making maternal cells and buding cells separately detected. Due to the fact that the budding cells are in the growing period, higher cell viability and non-specific esterase activity are possessed. At the same time, the non-specific esterase activity of sublethally injured cells is activated. Zhao et al. [44] found that non-specific esterase of *E*. *coli* were inactivated under stress of PEF, showing the difference inactivation mechanism of electric field in *E*.*coli* and *P*. *rhodanensis*.

**Table 1:** Percentage of *P*. *rhodanensis* cells staining and fluorescence intensity mean in P2 after MPEF in PBS at 80 pulses for 0-500V.

Voltage (V)	% Parent (P2)	Mean (M)
0	99.7	17252
100	100	28319
200	100	28435
300	100	28948
400	100	18266
500	100	17456

**Fig 8:**
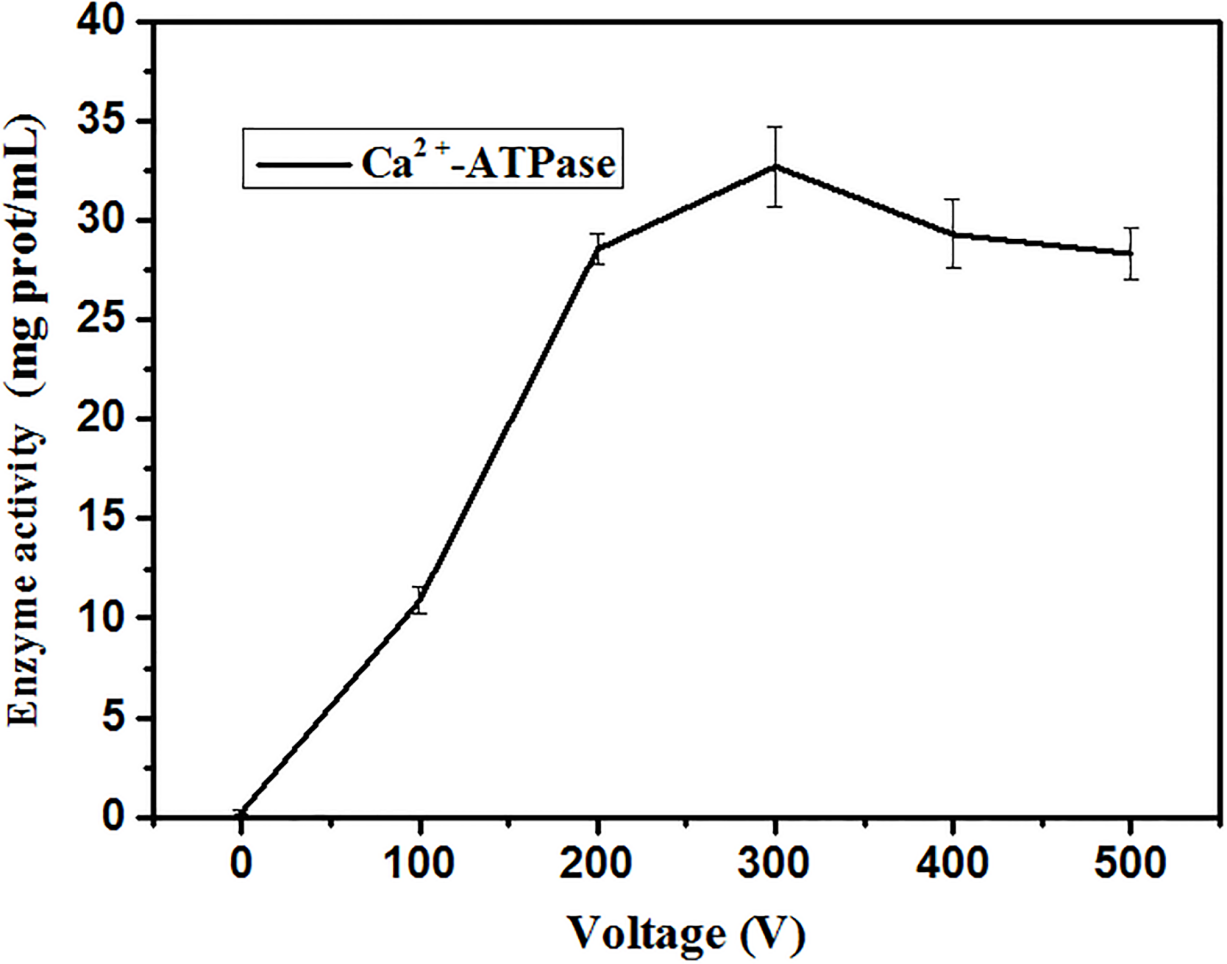
Ca^2+^-ATPase activities of *P*. *rhodanensis* exposed to MPEF for different voltage.

The cells were exposed to MPEF treatments at 80 pulses for 0 (A), 100 (B), 200 (C), 300 (D), 400 (E), 500 (F) V.Effects of MPEF treatment at 80 pulse for different voltage on the Ca^2+^-ATPase activities of plasma membrane were explored in this section. Fig 8 suggested that Ca^2+^-ATPase activities significantly increased after MPEF treatment, contrast to the untreated *P*. *rhodanensis* of only 0.25±0.09 mg prot/mL. When 300 V was applied to *P*. *rhodanensis*, the Ca^2+^-ATPase activities reached the highest level. This may be explained by the highest Ca^2+^-ATPase activities of sublethally injured cells under 300V, with further increase of voltage, cell damage gradually accumulated to the programmed death stage. Ca^2+^-ATPase presented in eukaryotic cells is responsible for fine-tuning the internal Ca^2+^ concentrations [53]. Therefore, the results suggested that MPEF treatment disturb the Ca^2+^ homeostasis in cells.

The change in activity of cellular enzymes of *P*. *rhodanensis* after MPEF at 80 pulses for 0 V, 200 V and 400 V was detected (Tables 2 and 3). Twelve intracellular enzymes activities were identified in untreated *P*. *rhodanensis* (100% as contrast), α-glucosidase, N-acetyl-β-glucosaminidase, Alkaline phosphatase and Acid phosphatase in *P*. *rhodanensis* were activated after MPEF treatment at 200V, while activity of other enzymes decreased in different degrees. Only seven cellular enzymes could be detectable when the voltage increases to 400 V, among them, the activity of five enzymes (Naphthol-AS-BI-phosphohydrolase, α-mannosidase, Leucine arylamidase, Esterase Lipase, Lipase) significantly decreased. Antioxidant enzymes, considered to be the defense against free radicals, preventing the occurrence of oxidative stress [54]. From Table 3, we can conclude that antioxidant enzyme activity in the *P*. *rhodanensis* cells was significantly reduced (P < 0.05) as the voltage increasing.

**Table 2:** Changes in intracellular enzymes activities of *P*. *rhodanensis* exposed to MPEF under 0, 200, 400V.

Species	Enzyme activity %		
	Control	200V	400V
Alkaline phosphatase	100±1.32	126.83±2.08	96.79±2.15
Acid phosphatase	100±1.89	107.36±2.32	81.46±1.98
Naphthol-AS-BI-phosphohydrolase	100±1.97	94.67±1.85	59.75±1.32
α-mannosidase	100±3.05	87.32±1.74	65.61±3.08
Leucine arylamidase	100±1.63	82.09±3.15	59.32±2.16
Esterase Lipase(C8)	100±1.19	66.54±4.11	8.96±3.1
Lipase(C04)	100±2.06	65.89±3.11	1.15±0.08
Valine arylamidase	100±2.35	63.24±2.78	0
Esterase(C4)	100±1.94	45.32±4.13	0
Cystine arylamidase	100±1.86	28.78±1.75	0
β-glucosidase	100±2.11	21.32±2.5	0
α-glucosidase	100±2.07	10.39±1.73	0
N-acetyl-β-glucosaminidase	0	100±2.07	0
α-glucosidase	0	100±1.75	0

**Table 3:** Changes in antioxidant enzyme activities of *P*. *rhodanensis* exposed to MPEF under 0, 200, 400V.

Species	Enzyme activity %		
	Control	200V	400V
Superoxide dismutase (SOD)	100±1.86	78.25±2.13	42.51±1.98
Catalase (CAT)	100±1.45	79.63±3.05	56.37±2.15
Glutathione peroxidase (GSH-Px)	100±1.93	87.32±2.27	55.93±3.41

Enzymes are biocatalysts that synthesized in vivo, all metabolic reactions in the organism cannot be carried out without enzymes. The decrease in enzyme activity represents that MPEF treatment affects the normal metabolism of *P*. *rhodanensis*, which may also be one of the reasons of cells’ death.

### Effect of MPEF on the proteins and nucleic acids of *P*. *rhodanensis*

**Fig 9:**
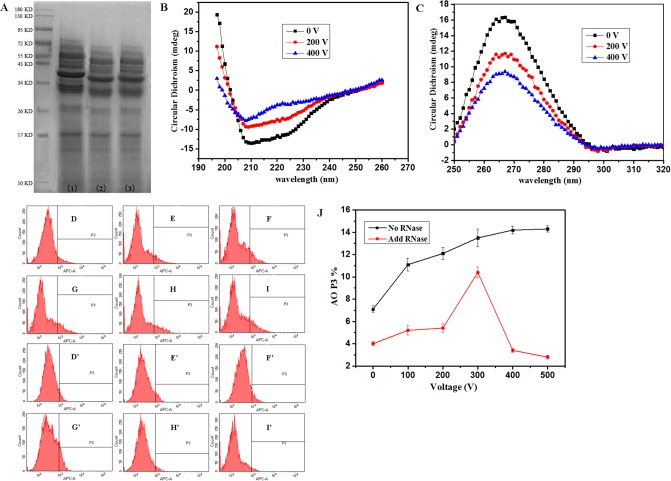
Changes in nucleic acid and protein structure of *P*. *rhodanensis* at different voltage. (A) SDS-PAGE pattern of intracellular proteins of *P*. *rhodanensis* exposed to MPEF at 80 pulses for 0 V(1), 200 V(2), 400 V(3). (B) Far-UV CD spectra of intracellular proteins of *P*. *rhodanensis* exposed to MPEF at 80 pulses for 0 V (black), 200 V (red) and 400 V (blue). (C) Near-UV CD spectra of nucleic acid of *P*. *rhodanensis* exposed to MPEF at 80 pulses for 0 V (black), 200 V (red) and 400 V (blue). The APC-A (red fluorescence collected at 633 nm) channel fluorescence histograms of *P*. *rhodanensis* stained by AO. The cells were exposed to MPEF treatments at 80 pulses for 0 (D, D’), 100 (E, E’), 200 (F, F’), 300 (G, G’), 400 (H, H’), 500 (I, I’) V. D-I represents the cells without RNase, D’-I’ represents the cells added with RNase. (J) Percentage of *P*. *rhodanensis* cells in P3 after MPEF in PBS at 80 pulses for 0-500V, the black line represent non-RNase-treated samples and the red line represent RNase-treated samples.

Fig 9A showed the difference of SDS-PAGE of total proteins between the MPEF-treated cells and original cells. As shown in Fig 9A, the strip of proteins by MPEF treatment were slightly moved down compared with the untreated cells, and the type of protein had no obvious change, indicating that the primary structure of intracellular proteins were not destroyed obviously, the inactivation of *P*. *rhodanensis* by MPEF may be caused by the changes of protein secondary structure. The changes of secondary structure of intracellular protein were studied under the same MPEF treatment conditions. The CD spectra of intracellular protein before and after MPEF treatment were shown in Fig 9B. They had double negative peaks in the far-UV CD spectra at around 208 and 222 nm, which are the features of α-helix [55]. Similarly, negative peaks at around 215 nm were detected, demonstrating β-sheet also exist in the *P*. *rhodanensis* protein. In addition, compared with the untreated sample, the content of α-helix and β-sheet of the MPEF-treated samples were gradually diminished. These indicate that MPEF treatment not only promote the leakage of intracellular protein, but also cause protein denaturation of *P*. *rhodanensis*, all of these will result in cells death.

Near-UV CD spectra can be used to reflect nucleic acid changes, results were shown in Fig 9C. The positive peak at 277 nm is generated by the accumulation of bases [56]. Compared to the untreated samples, the positive peaks were significantly reduced when different voltage were applied to the samples, representing the conformation of the nucleic acid has changed. Besides, the CD peak position had basically no changed, demonstrating that MPEF only makes base stacking and double helix structure become loose, whether it result in unwinding also need to be explored.

The change in APC-A channel fluorescence histograms of *P*. *rhodanensis* cells stained by AO can be seen in Fig 9(D)–9(I) and 9(D’)–9(I’) and Table 4, and cells in P3 region represent the proportion of single-stranded DNA and RNA. The fluorescence intensity mean of MPEF-treated cells were shifted toward higher channel numbers as the voltage increased from 0 to 200V, and then began to drop when the voltage continued to rise. Meanwhile, P3% was continuous increasing (Fig 9J, black line). Therefore, MPEF treatment may cause DNA unwinding and RNA break into small fragments, resulting in a sustained increase in P3% and the change of fluorescence intensity. The results suggest that nucleic acids was one of the objective for MPEF induced damage.

**Table 4:** Fluorescence intensity mean in P3 after MPEF in PBS at 80 pulses for 0-500V.

Mean	Voltage (V)					
	0	100	200	300	400	500
No RNase	1047	1676	2096	1954	1846	1403
Add RNase	1202	1499	1344	1208	1432	1552

In order to analyze the effect of MPEF treatment on DNA and RNA individually, RNase was added to samples [57]. After digestion of RNase, RNA in the *P*. *rhodanensis* cells was total eliminated. Therefore, the Fig D’-I’ only reflected the variation of DNA induced by MPEF treatment. As shown in Fig 9D–9I and Table 4, no significant changes could be observed in fluorescence intensity mean, the only difference was the number of cells located in R3 region after MPEF treatment. The red line in Fig 9J represents the proportion of single-stranded DNA, showing a tendency of increase firstly (0-300V) and then decrease rapidly, finally, P3% was lower than the control group. These demonstrate that part of the double-stranded DNA unwinding to single-stranded DNA when the voltage is low, and continuing to increase the voltage, MPEF treatment can also destroy single-stranded DNA, resulting in a decrease in its event. Based on the results, MPEF treatment can produce destructive effect on RNA and DNA, causing cell death.

### Effect of MPEF on the ultrastructure and mitochondrial membrane potential of *P*. *rhodanensis*

**Fig 10:**
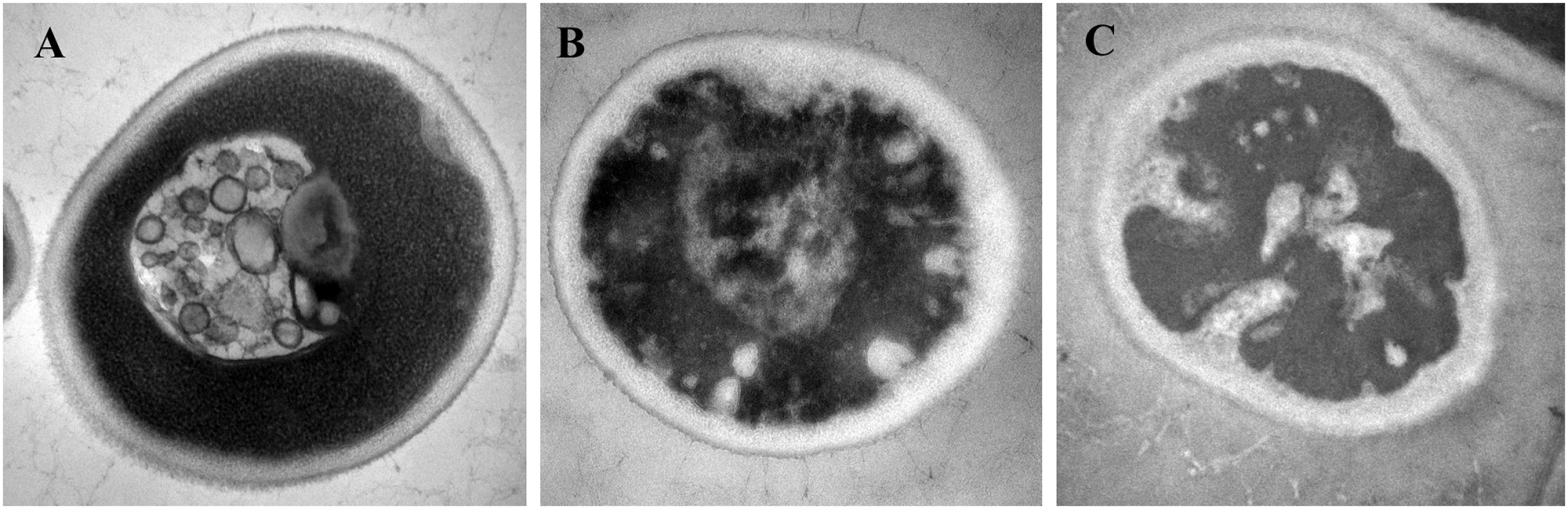
TEM images of the *P*. *rhodanensis* cells: (A) untreated cells; MPEF-treated cells: (B) 200V, (C) 400V. Arrows represent visible changes on the cell ultrastructure.

The ultrastructure structure of MPEF-treated *P*. *rhodanensis* cells was observed by TEM. Fig 10A demonstrates the regular shape and morphology of untreated cells, with well-defined cell membrane, complete and compact intracellular structure and evenly distributed cytoplasm. However, the cells of MPEF-treated for 200V began to appear depression, the cytoplasm was gradually pycnotic, and organelles become bad to identify (Fig 10B), which was consistent with the observation of SEM. Moreover, when treatment voltage was 400V (Fig 10C), serious damages on inner cell components were observed, such as unclear membrane structure, plasmolysis, undistinguishable organelles and turbid cytoplasm. In sum, MPEF treatment converted the compact ultrastructure of cells into loose ones and then broke them.

**Fig 11:**
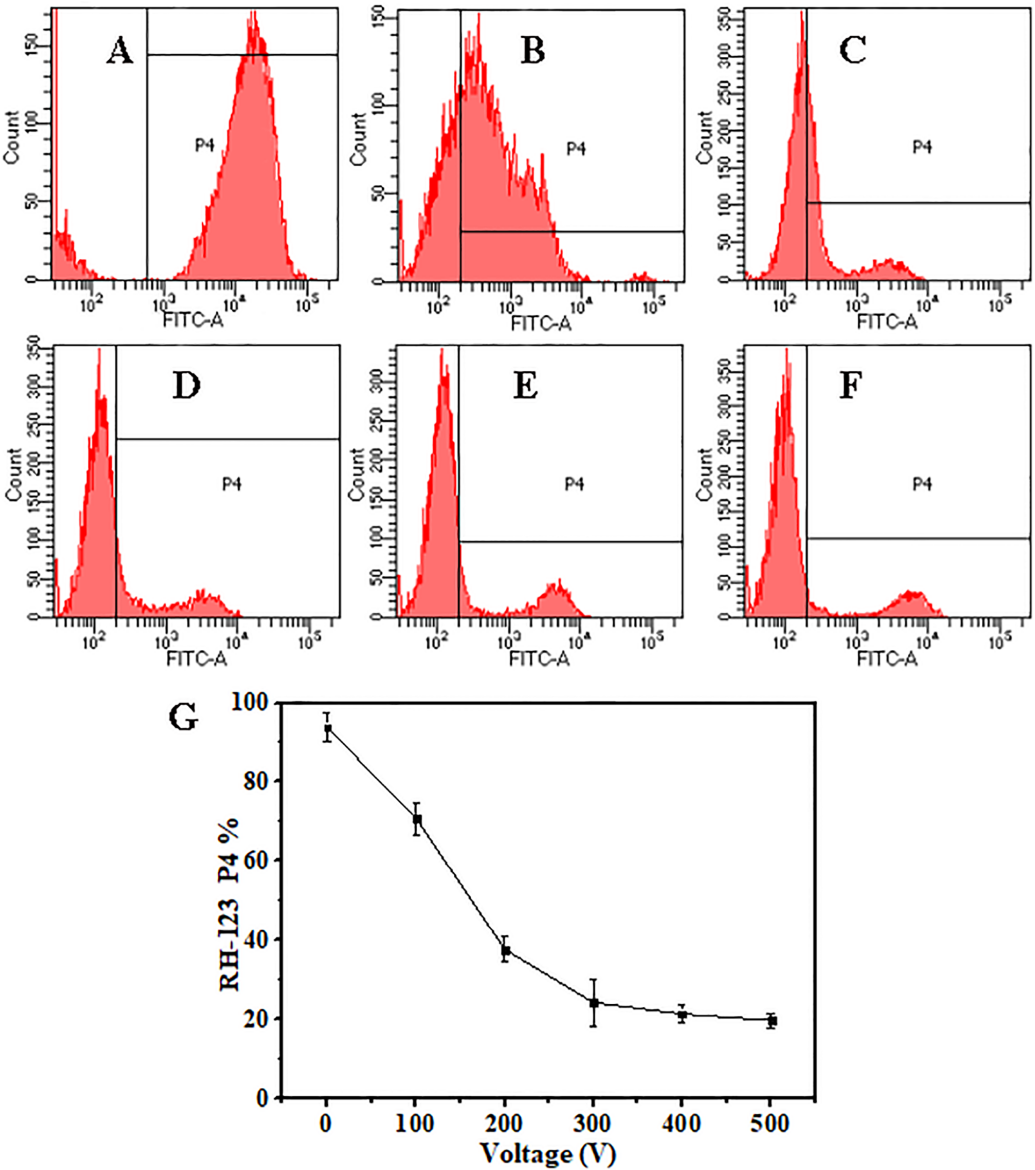
The FITC-A (green fluorescence collected at 488 nm) channel fluorescence histograms of *P*. *rhodanensis* stained by RH-123.

Flow cytometry histograms of *P*. *rhodanensis* cells stained with RH-123 before and after MPEF treatment were shown in Fig 11(A)–11(F). Cells in P4 region are marked by RH-123, others are autofluorescence. Fig 11G displayed the percentage of RH-123 stained *P*. *rhodanensis* cells (P4%) exposed to MPEF under different voltage. Most cells (93.8%) without MPEF treatment were located at R4 region, indicating a plenty of cells with intact membrane and normal transmembrane potential. There were apparent decrease of P4% with the increasing of voltage (Fig 11G), and only 19.8% cells were in the P4 region under 400 V, representing MPEF had a great effect on mitochondrial membrane potential, and the transmembrane potential was seriously damaged with the increasing of voltage, which was consistent with PI staining results.

The cells were exposed to MPEF treatments at 80 pulses for 0 (A), 100 (B), 200 (C), 300 (D), 400 (E), 500 (F) V. (G) Percentage of *P*. *rhodanensis* cells in P4 after MPEF in PBS at 80 pulses for 0-500V.

## Conclusions

The main objective of this research was to study the potential of MPEF to inactivate *P*. *rhodanensis*, and then explore the underlying mechanism. Experimental investigations showed that the achieved maximum inactivation was 6.48± 0.03 log_10_ cycles at 500V and 80 pulses. After 200 V and 80 pulses, the rate of sublethal injury cells reached maximum up to 27.2%, cell membrane damage increased with voltage, meanwhile, membrane fluidity is declining. The leakage of protein, nucleic acid and K ^+^, Mg^2 +^ are positively correlated with voltage.

After MPEF treatment, Ca^2+^ homeostasis was destroyed, non-specific esterase activity has a slight increase, and on the contrary, there is a clear decline of intracellular enzymes and antioxidant enzyme activity. The effect of MPEF on protein structure was realized by changing its secondary structure, on the other hand, double helix structure of DNA become loose and unwinding after 400V MPEF treatment. Besides, cell appeared plasmolysis and missing organelles, and transmembrane potential apparent decrease with the increasing of voltage. Therefore, MPEF can effectively inactivate *P*. *rhodanensis* by cell membrane and ultrastructure damage, intracellular compounds leakage, the reducing of cellular enzyme activity and transmembrane potential, and the change in protein and nucleic acid structure.
